# Case Report: Dermoscopic and pathologic features of primary cutaneous follicle center lymphoma involving the nasal ala

**DOI:** 10.3389/fonc.2026.1767218

**Published:** 2026-07-22

**Authors:** Yang Han, Sheng-nan Zhao, Xinhuan Wang, Song Zheng

**Affiliations:** 1 Department of Dermatology, Affiliated Central Hospital of Shenyang Medical College, Shenyang, Liaoning, China; 2 Department of Dermatology, The First Hospital of China Medical University, Shenyang, Liaoning, China

**Keywords:** B-cell lymphoma, cutaneous lymphoma, histopathology, primary cutaneous follicle center lymphoma, radiotherapy

## Abstract

**Background:**

Primary cutaneous follicle center lymphoma (PCFCL) is a rare, indolent B-cell lymphoma that typically presents as asymptomatic lesions on the scalp, forehead, or trunk, with disease confined to the skin. Involvement of the nasal region is highly uncommon and prone to misdiagnosis because of its nonspecific clinical features.

**Case description:**

An 84-year-old woman presented with a collection of asymptomatic, firm, agminated papules on the left nasal ala, first noted about 2 years prior. At dermoscopy, the lesions presented fine, linear, irregular vessels on a salmon-colored background. The histopathological evaluation revealed nodular lymphoid infiltrates within the dermis, a prominent grenz zone, and well-demarcated lymphoid follicles composed of atypical lymphocytes with a thin mantle zone. The immunohistochemical analysis showed diffuse positivity for CD20, BCL6 and CD10, and negativity for BCL2 and CD5 in the atypical cells, which were surrounded by a thin layer of CD3-positive reactive T lymphocytes. The diagnosis of PCFCL was established on the basis of the clinical, dermoscopic, and histopathologic findings, and curative radiation therapy was initiated.

**Conclusion:**

PCFCL involving the nasal ala is extremely rare and presents with nonspecific dermoscopic patterns that may delay or obscure diagnosis. Accurate identification relies on the integration of dermoscopic assessment, histopathological examination, and immunohistochemical profiling.

## 1 Introduction

Primary cutaneous follicle center lymphoma (PCFCL) is the most common subtype of primary cutaneous B-cell lymphoma (CBCL), accounting for approximately 50% of all CBCL cases (1). It is characterized by disease confined to the skin without evidence of systemic involvement at initial presentation. PCFCL was first recognized as a distinct neoplastic entity in the 2008 World Health Organization (WHO) Classification of Haematolymphoid Tumours (2) and was further characterized in the 2018 WHO-EORTC classification for primary cutaneous lymphomas, which highlights its indolent clinical behavior and distinct clinicopathological features (3).

PCFCL predominantly affects middle-aged and older adults, shows no significant sex predilection, and is rarely diagnosed in children (4). Clinically, it may present as solitary or clustered indurated erythematous papules, plaques, nodules, or tumors, which are typically non-ulcerated and asymptomatic. Lesions most frequently occur on the scalp, forehead, and trunk, whereas involvement of the lower extremities is unusual and nasal involvement is exceedingly rare (5). The presence of pink agminated papules at atypical sites, such as the nasal ala, may further complicate the diagnostic process.

The current report describes an elderly woman with PCFCL presenting as asymptomatic, firm, agminated papules on the left nasal ala. Because of the rarity of nasal PCFCL and the diagnostic challenges posed by its presentation, dermoscopic, histopathological, and immunohistochemical features are discussed to strengthen clinical recognition and improve diagnostic accuracy for this uncommon manifestation of PCFCL.

## 2 Case report

An 84-year-old woman presented with a 2-year history of asymptomatic agminated papules on the left nasal ala. Her past medical history was unremarkable, and she reported no preceding trauma or insect bites. Physical examination revealed several pink, translucent, firm, clustered papules on the left nasal ala (**Figure 1A**), without associated fever or regional lymphadenopathy. Laboratory studies, including complete blood count, erythrocyte sedimentation rate, C-reactive protein, and a comprehensive metabolic panel, were all within normal reference ranges.

**Figure 1:**
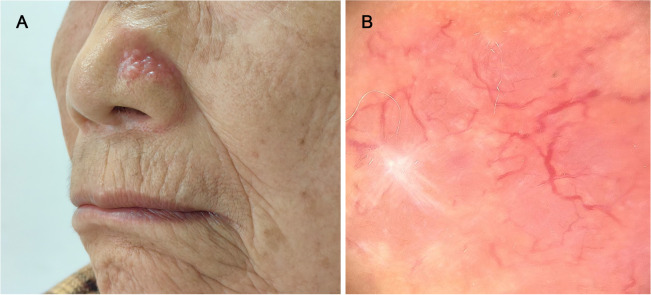
Clinical and dermoscopic features of the lesion. **(A)** Clustered pink, firm papules on the left nasal ala of an elderly woman. **(B)** Dermoscopic examination showing linear and arborizing vessels on a salmon-colored background.

Dermoscopy demonstrated linear and arborizing vessels on a salmon-colored background (**Figure 1B**). A skin biopsy was performed for further diagnostic evaluation. Histopathologic examination revealed nodular lymphoid infiltrates confined to the dermis with a prominent grenz zone (**Figure 2**). High-power microscopy showed well-formed lymphoid follicles composed of atypical lymphocytes with a thin mantle zone, only scattered plasma cells were identified throughout the specimen. Immunohistochemical staining demonstrated diffuse positivity for CD20, BCL6 and CD10, within the atypical lymphoid population, with a thin rim of CD3-positive reactive T lymphocytes surrounding the neoplastic follicles. The Ki-67 proliferation index was quantified as 30% (**Figure 3**). These findings confirmed the diagnosis of primary cutaneous follicle center lymphoma.

**Figure 2:**
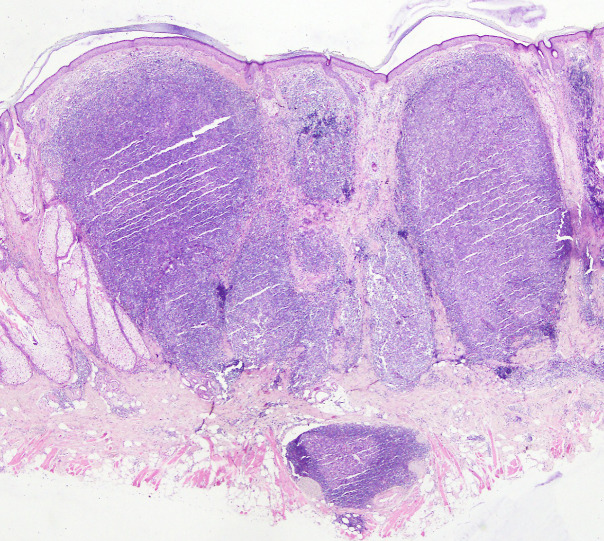
Histopathologic findings. Skin biopsy showing a nodular dermal lymphoid infiltrate with a prominent grenz zone (hematoxylin-eosin stain).

**Figure 3:**
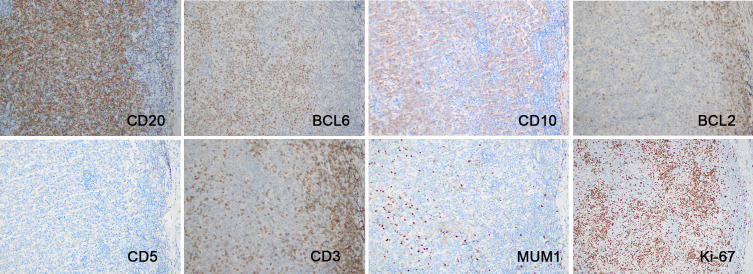
Immunohistochemical profile of the lymphoid infiltrate (×200). Atypical lymphoid cells showing diffuse positivity for CD20, BCL6 and CD10, negativity for BCL2 and CD5, and a surrounding thin rim of CD3-positive reactive T lymphocytes, with only scattered cells exhibiting immunoreactivity for MUM-1. The Ki-67 proliferation index was quantified as 30% (×100).

Surgical excision was unsuitable because of the patient’s advanced age and the anatomic location on the nasal ala, and curative radiation therapy was initiated using a 6 MV electron beam. A fractional dose of 2 Gy was delivered five days per week with a two-day weekly break, for a total of 15 fractions and an overall cumulative radiation dose of 30 Gy. The lesions regressed substantially within one month, with complete remission by three months. No recurrence was observed at the 1-year follow-up.

## 3 Discussion

The characteristic heterogeny of cutaneous lymphomas is evinced across subtypes by molecular alterations, differences in cellular origin, tumor morphology, and immunophenotype and by the clinical behavior and therapeutic response of the tumors (6). Among primary cutaneous lymphomas, T-cell lymphomas predominate, representing approximately 75% to 80% of cases, whereas primary CBCLs account for only 20% to 25% of cases (7). According to the fifth edition of the WHO classification of hematolymphoid tumors and the International Consensus Classification of mature lymphoid neoplasms, CBCLs can be divided into five major subtypes, including PCFCL; primary cutaneous marginal zone B-cell lymphoma (PCMZL); primary cutaneous diffuse large B-cell lymphoma, leg type (PCDLBCL-LT); intravascular large B-cell lymphoma; and Epstein–Barr virus–positive mucocutaneous ulcer (6). Among these, PCFCL is the most common, accounting for roughly half of all CBCLs. While the etiology remains unclear, occasional associations with Borrelia burgdorferi, hepatitis C virus, and human herpesvirus 8 have been reported. However, a causal relationship has not been definitively established (8).

PCFCL predominantly affects middle-aged and older adults, with no significant sex predilection, and is exceedingly rare in children. Clinically, it presents as asymptomatic, firm, localized erythematous papules, plaques, nodules, or tumors that are typically non-ulcerative (5). Lesions most commonly arise on the scalp, forehead, and trunk, are seldom found on the lower extremities, and generally remain confined to a single anatomical region, with extracutaneous spread being uncommon. Our patient’s age at diagnosis is not inconsistent with the age demographic typically affected by PCFCL. However, involvement of the nasal ala is exceptionally rare and so emphasizes the broad clinical spectrum of PCFCL and the potential for diagnostic delay, particularly when lesions arise at uncommon sites.

Histopathologically, PCFCL is characterized by dermal lymphoid infiltrates that may extend into the subcutaneous tissue in larger nodules, typically sparing the epidermis and exhibiting a prominent grenz zone. Depending on the pattern of infiltration, PCFCL can be classified into follicular, combined follicular and diffuse, and diffuse growth patterns (1). The neoplastic follicular centers are composed of variable proportions of centrocytes and centroblasts arranged in nodular to diffuse configurations. The follicles generally lack polarization into light and dark zones and contain few or no tangible body macrophages. The histologic features may vary according to lesion duration, growth rate, and anatomical location. Follicular growth patterns are more frequently observed in scalp lesions than in those on the trunk, and early, small lesions tend to consist predominantly of small centrocytes with a follicular pattern, whereas more advanced lesions, such as large nodules or tumors, often show an increased proportion of large neoplastic cells, particularly large centrocytes and cleaved cells, with only a limited number of centroblasts that do not form confluent sheets (3, 6).

The nodular dermal lymphoid infiltrates with a prominent grenz zone described at histopathology in the present case, as well as the high-power microscopy revealing lymphoid follicles composed of atypical lymphocytes with a thin mantle zone, are all consistent with the classic histopathologic features of PCFCL.

Immunophenotypic profiling is essential for establishing a definitive diagnosis of PCFCL. Neoplastic cells uniformly express B-cell lineage markers, including CD19, CD20, CD22, CD79a, and BCL6. CD10 expression is variable and tends to be observed more frequently in follicular growth patterns than in diffuse patterns (4, 5, 9). Importantly, the neoplastic cells typically lack BCL2 expression or exhibit only weak positivity, and they are generally negative for MUM1, CD5, and CD43. A Ki-67 proliferation index of<50% is often noted (10).

The tumor cells in the present case showed diffuse immunopositivity for CD20 and BCL6 within the atypical lymphoid population, accompanied by a thin rim of CD3-positive reactive T lymphocytes. These findings are consistent with the characteristic immunophenotype of PCFCL, and provided essential diagnostic support.

The most commonly described feature of PCFCL at dermoscopy is the presence of fine, linear, irregular vessels on a salmon-colored background (11). However, the combination of pink agminated papules such as were mentioned here with nonspecific vascular patterns can complicate the clinical assessment, particularly in sun-exposed regions such as the nasal ala. In this setting, key differential diagnoses include nodular basal cell carcinoma (nBCC), nodular amyloidosis (NA), and granuloma faciale (GF).

nBCC typically manifests as pink, pearly nodules with surface telangiectasia and characteristic “rolled” borders (12). nBCCs grow slowly and preferentially arise on sun-exposed sites, with a notable predilection for the nasal region (13). Dermoscopic evaluation often reveals arborizing vessels as the predominant feature, followed by blue-gray ovoid nests and overall translucency (14). Histopathologically, nBCC is characterized by nodular aggregates of basaloid cells with peripheral palisading that extend from the epidermis into the dermis, separated from the surrounding mucinous and fibrotic stroma by prominent retraction clefts.

NA is a rare variant of primary cutaneous amyloidosis characterized by asymptomatic, slowly progressive, erythematous, waxy yellowish tumor-like nodules that commonly affect the face, neck, trunk, and extremities. Some lesions exhibit a semi-translucent appearance. In contrast to PCFCL, dermoscopic evaluation of NA typically reveals a homogeneous orange to orange-pink background interspersed with small white structureless areas. Occasional hemorrhagic spots and linear, curved, or telangiectatic vessels may be present, but arborizing vessels are generally absent (15). Histopathologically, NA demonstrates deposition of dense amorphous eosinophilic material within the dermis and vascular walls, often accompanied by hemorrhage and variable numbers of plasma cells. Amyloid deposits stain brick-red with Congo red, and plasma cells may show kappa or lambda light-chain restriction, supporting their role as the source of amyloid material.

GF is another important differential diagnosis. GF is a rare, chronic form of leukocytoclastic vasculitis that most commonly involves the forehead, nose, and cheeks (16). Clinically, lesions appear as single or multiple erythematous nodules or plaques. Dermoscopically, GF may resemble PCFCL, displaying a yellowish to reddish background and diverse vascular patterns. However, several features are more characteristic of GF, including dilated follicular openings, perifollicular whitish halos, follicular keratotic plugs, and numerous rosettes (17). Histopathologic examination typically reveals a dense polymorphous inflammatory infiltrate composed of neutrophils with nuclear debris, lymphohistiocytes, eosinophils, and plasma cells. A well-defined grenz zone is often present beneath the epidermis and around hair follicles. Fibrin deposition within vascular walls with adjacent hemorrhage may also be seen, reflecting underlying vasculitic changes.

PCMZL, as a distinct subtype of primary CBCLs, which frequently exhibits characteristics overlap with PCFCL (18). PCMZL accounts for approximately 25% to 30% of primary CBCLs and shows a male predilection, typically affecting patients in their 50s to 60s. As an indolent B-cell lymphoma, it generally presents as asymptomatic or mildly pruritic, solitary or clustered pink-to-red papules, plaques or nodules, mainly occurring on the arms and trunk. The histopathological manifestation of PCMZL is nodular or diffuse dermal infiltration of lymphocytes such as small lymphocytes, marginal zone B cells and plasma cells, accompanied by a minor population of reactive T lymphocytes and scattered eosinophils, and occasionally infiltrating the subcutaneous tissue and fatty layer. The immunophenotypic profile is distinctive, showing negative for CD5, CD10 and BCL6, positive for CD20, CD79a, PAX5 and BCL2 (19). Dermoscopically, serpentine vessels and unfocused linear vessels can be seen against a salmon-colored background in PCBCL lesions (20).

PCFCL carries an excellent prognosis, with a 5-year disease-specific survival rate exceeding 95%. Although multiple therapeutic modalities have been utilized, no standardized treatment algorithm has been established. Management is guided by factors such as the number, size, and anatomical location of lesions, as well as individual patient characteristics. Surgical excision is preferred for small, localized lesions. For patients who are not suitable surgical candidates, definitive radiation therapy represents an effective alternative, as demonstrated in the present case. Radiotherapy to the nasal ala may trigger cutaneous erythema, desquamation, nasal dryness, hypopigmentation, telangiectasia, and even tissue necrosis. These adverse risks were minimized via the application of 6 MV electron beams, accurate lesion positioning and standardized radiation shielding. The patient only developed transient erythema and mild nasal dryness throughout treatment, and all these symptoms fully resolved by the 1-year follow-up. In individuals with more widespread disease, systemic rituximab has shown substantial efficacy. In the rare event of extracutaneous dissemination, multi-agent chemotherapy may be warranted (21).

Follow-up protocols for PCFCL are individualized according to clinical manifestations. Semi-annual surveillance incorporating comprehensive cutaneous inspection and lymph node palpation is generally recommended (19), and dermoscopy serves as a valuable auxiliary tool for identifying subtle early recurrent lesions. Consistent with this individualized surveillance principle, close follow-up was performed in the present patient. Post-treatment re-evaluations were conducted at 1 month, 3 months, and 1 year, and long-term regular follow-up will be continued thereafter.

## 4 Conclusion

PCFCL is a rare cutaneous lymphoma with nonspecific clinical and dermoscopic features, underscoring the critical role of histopathological examination and immunohistochemical profiling in establishing an accurate diagnosis, particularly in uncommon anatomical locations such as the nasal ala. Definitive radiotherapy can be an effective treatment in individuals or cases that are not suitable for surgical excision, often resulting in marked regression. Long-term regular follow-up is important for prompt detection of possible recurrence and for monitoring systemic status to exclude the development of extracutaneous lymphoma.

## Data Availability

The original contributions presented in the study are included in the article/supplementary material. Further inquiries can be directed to the corresponding author.
